# Reactive oxygen species and serum antioxidant defense in juvenile idiopathic arthritis

**DOI:** 10.1007/s10067-014-2571-9

**Published:** 2014-03-22

**Authors:** Joanna Lipińska, Stanisława Lipińska, Jerzy Stańczyk, Agata Sarniak, Anna Przymińska vel Prymont, Marek Kasielski, Elżbieta Smolewska

**Affiliations:** 1 Department of Pediatric Cardiology and Rheumatology, Second Chair of Pediatrics, Medical University of Lodz, 36/50 Sporna St, 91-738 Lodz, Poland; 2 Department of General Physiology, Chair of Experimental and Clinical Physiology, Medical University of Lodz, Mazowiecka 6/8, 92-215 Lodz, Poland; 3 Outpatient Department of Pediatric Rheumatology, Maria Konopnicka’ Memorial Hospital Lodz, Lodz, Poland; 4 Center of Medical Education, Practical Training Center, Medical University of Lodz, Lodz, Poland

**Keywords:** Ferric reducing ability of plasma (FRAP), Juvenile idiopathic arthritis (JIA), Nitrates (NO_3_^−^), Nitric oxide (NO), Nitrites (NO_2_^−^), Thiobarbituric acid-reactive substances (TBARs)

## Abstract

In autoimmune inflammatory diseases, including juvenile idiopathic arthritis (JIA), which leads to joint destruction, there is an imbalance between production of reactive oxygen species (ROS) and their neutralization which, as a consequence, leads to “oxidative stress.” The aim of the study was to assess the concentration of oxidative stress markers: nitric oxide (NO), a degree of lipid membrane damage, and total antioxidant plasma capacity in children with JIA. Thirty-four children with JIA were included into the study. A degree of lipid membrane damage (lipid peroxidation products) was estimated as thiobarbituric acid-reactive substances (TBARs), NO concentration as NO end-products: nitrite/nitrate (NO_2_^−^/NO_3_^−^) and total antioxidant plasma capacity as ferric reducing ability of plasma (FRAP). NO_2_^−^/NO_3_^−^ serum concentration in children with JIA was statistically significantly higher than that in healthy children (*p* = 0.00069). There was no significant difference in TBAR levels between children with JIA and the control group. FRAP in sera of children with JIA was lower than that in healthy children, but the difference was not statistically significant. A statistically significant positive correlation was observed between NO end products and the 27-joint juvenile arthritis disease activity score (JADAS-27) and ESR, and a negative correlation was observed between FRAP and C-reactive protein (CRP) and white blood cell count (WBC). Our results confirm the increased oxidative stress in children with JIA. Overproduction of NO and decrease in the antioxidant plasma capacity may be involved in JIA pathogenesis.

## Introduction

*Juvenile idiopathic arthritis* (JIA) is a heterogeneous group of autoimmune diseases of unknown origin, with onset before the age of 16 years. JIA is considered to be the most common connective tissue disease of childhood. It is characterized by leukocyte infiltration in the synovium leading to a chronic inflammation in the joints, which persists for more than 6 weeks, with consequent destruction of articular tissue [1, 2]. Recently, considerable interest has been generated in determining the JIA pathogenesis, looking for new therapeutic strategies and for the prognostic factors, which would permit an early identification of patients with a poor prognosis, justifying the application of an aggressive treatment in the early stages of the disease [2, 3].

Reactive oxygen species (ROS) play a pivotal role in physiological processes. ROS mediate and regulate many cell functions, including cytokine production, signal transduction, mitochondrial functions, immune processes, gene expression, and apoptosis. The effects of ROS depend on their serum concentration. Nitric oxide (NO), a unique biological messenger molecule, is a short-lived gaseous free radical. It is synthesized from l-arginine by NO synthase in various cell types such as chondrocytes, synoviocytes, and leukocytes, e.g., phagocytic neutrophils. Direct measurement of NO and other free radicals in standard laboratories is difficult because of their biochemical instability. Due to the above, concentrations of NO end-products nitrite/nitrate (NO_2_^−^/NO_3_^−^) or the activity of NO synthase is usually evaluated. NO is involved in immune regulation, neurotransmission, and vasodilatation. Recently, the imbalance between formation of ROS and body antioxidant defense in homeostasis disturbances is widely discussed [4]. The currently reported data demonstrated a significant role of ROS in the etiopathogenesis of autoimmune diseases. In chronic inflammatory processes, after long stimulation, the exhaustion of the body’s antioxidative reserves results in a harmful activity of ROS, as they gain an advantage over the antioxidant system—this phenomenon is called “oxidative stress” [5].

Many studies are conducted on the role of ROS in the etiopathogenesis of rheumatoid arthritis (RA), which seem to be a main direct factor destroying the joint tissues, but in the pediatric population with JIA, their role has still not been elucidated [5–9]. It could be hypothesized that patients with JIA have defective defense mechanisms against ROS and these mechanisms vary according to the JIA subtypes.

The aim of the study was to assess markers of the oxidative stress: concentrations of NO end-products nitrite/nitrate (NO_2_^−^/NO_3_^−^), thiobarbituric acid-reactive substances (TBARs), and total antioxidant capacity of plasma via ferric reducing ability of plasma (FRAP) in the sera of children with JIA in comparison with healthy children. Moreover, the study also aimed to correlate these markers with disease characteristics (type of JIA onset, disease activity).

## Patients and methods

### Patients

Thirty-four children (22 girls, 12 boys) with JIA were included into the study. The patients were entered in regard to the 2001 International League Against Rheumatism (ILAR) classification criteria [10]. Twenty-six children had polyarticular JIA type of onset, and eight had oligoarticular JIA. They were aged 7–18 years (mean 13.8 ± 1.2 years, range 7–18), had disease duration time of 0.5–7.5 years (mean 3.5 ± 0.8 years), and were treated with DMARDs (methotrexate, 28 patients or sulfasalazine, 6 patients) and low doses of steroids (prednisone <10 mg/day). All of the study group children were biologically naive (Table 1).

**Table 1:** Demographic, clinical, and laboratory parameters of children with JIA

Characteristics of children with JIA (*n* = 34)	
Female/male, *n* (%)	22/12 (64.7/35.3)
Age (years)/range	13.8 ± 1.2/7–18
Type of JIA onset	
Polyarticular, *n* (%)	26 (76.5)
IgM-RF-negative	24
IgM-RF-positive	2
Oligoarticular, *n* (%)	8 (23.5)
Persistent oligoarticular JIA	7
Extended oligoarticular JIA	1
Disease duration time (years)	3.5 ± 0.8
ESR (mm/h)	19.4 ± 20.5 (median 10)
ESR above cutoff value (>20 mm/h), *n* (%)	8 (23.5)
CRP (mg/dL)	2.67 ± 6.26 (median 0.39)
CRP above cutoff value (>0.5 mg/dL), *n* (%)	14 (41.2)
IgM-RF positivity (≥24 RU/mL), *n* (%)	2 (5.9)
Disease activity	
JADAS-27	23.94 ± 10.10 (median 25)

Data are means ± standard deviation (SD), unless otherwise indicated

*n* number of children, *ERS* erythrocyte sedimentation ratio, *CRP* C-reactive protein

Twenty sex- and age-matched healthy children (without autoimmune diseases) from the control group were also examined.

Serum samples (3 mL) were obtained simultaneously with routine laboratory tests (white blood cell (WBC) count, red blood cell (RBC) count, thrombocyte count, hemoglobin, C-reactive protein (CRP)). The activity of the rheumatoid process was assessed according to the 27-joint juvenile arthritis disease activity score (JADAS-27) [11]. Each patient was evaluated for the physician’s global assessment of overall disease activity, which was measured on a 10-cm visual analog scale (VAS; 0 = no activity and 10 = maximum activity); parent’s global assessment of the child’s overall well-being, which was measured on a 10-cm VAS (0 = very good and 10 = very poor); and 27-joint count including the cervical spine, elbows, wrists, metacarpophalangeal joints (from the first to the third), proximal interphalangeal joints, hips, knees, ankles, and ESR.

The study was approved by the local ethical committee. In every case, a written informed consent was obtained from the patient and parents before study entry.

### Methods

#### NO assay

The concentration of NO was assessed with an indirect method by measurement of NO end-products nitrite/nitrate (NO_2_^−^/NO_3_^−^) (Nitrate/Nitrite Colorimetric Assay Kit, Sigma-Aldrich Chemie GmbH, Switzerland). Total NO_2_^−^/NO_3_^−^ concentrations in the serum samples were determined by microcolorimetric Griess reaction for nitrite [12] following nitrate reductase-mediated nitrate ion reduction [13]. The absorbance of the chromophore was read at a wavelength of *λ* = 548 nm.

#### Determination of TBARs

Measurement of lipid peroxidation products was based on thiobarbituric acid-reactive substance (TBAR) concentration in plasma. Briefly, 1 mL of 0.05 mol/L H_2_SO_4_ and 0.5 mL of 1.23 mol/L trichloroacetic acid (TCA) were added to 0.02 mL of plasma, mixed, and then centrifuged for 10 min (1,500 × *g*, 20 °C). Fluorescence was measured at an excitation wavelength of *λ* = 515 nm and emission wavelength of *λ* = 546 nm.

#### FRAP assay

The total antioxidant plasma capacity was assayed in terms of the ferric reducing ability of plasma (FRAP) according to the original description by Benzie and Strain [14]. This method measures the ability of antioxidants contained in a sample to reduce ferric tripyridyltriazine (Fe^3+^-TPTZ) to a ferrous form (Fe^2+^-TPTZ) that absorbs light at *λ* = 593 nm.

### Statistical analysis

Data was expressed as mean and standard deviation (±SD). Results were analyzed using nonparametric Mann-Whitney tests, and correlation was found by the Spearman coefficient. Confidence level was 95 % and statistical significance was considered at a level of 0.05.

## Results

The demographic, clinical, and laboratory features of children with JIA are shown in Table 1.

### NO assay/concentration of NO_2_^−^/NO_3_^−^

**Fig. 1:**
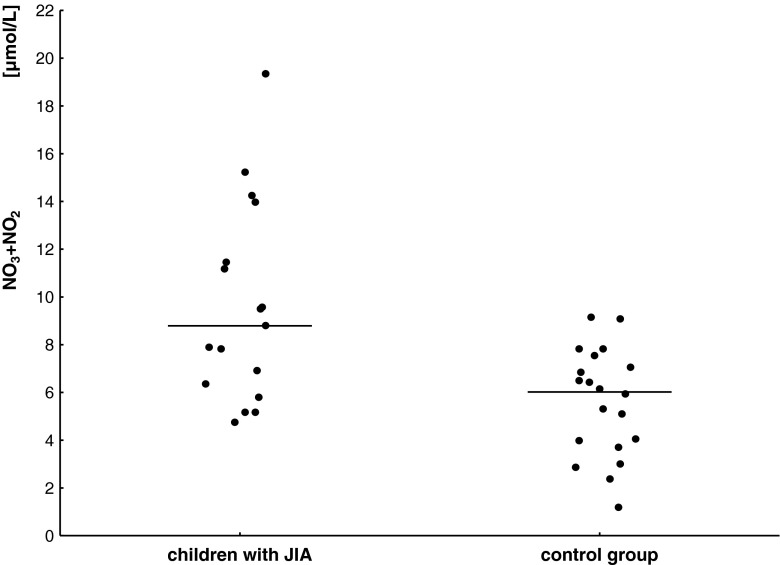
Nitrite/nitrate (NO_2_^−^/NO_3_^−^) serum concentration and mean value (*horizontal line*) in children with JIA and in the control group

Figure 1 shows higher serum concentrations of NO end-products and mean obtained from children with JIA compared to 20 healthy children.

Total NO_2_^−^/NO_3_^−^ concentrations in the serum samples of 34 children with JIA were significantly higher (9.59 ± 4.14 μmol/L) than those measured in sera of 20 sex- and age-matched children from the control group (5.59 ± 2.25 μmol/L; *p* = 0.00069). There was no statistically significant difference in serum NO_2_^−^/NO_3_^−^ concentrations between children with polyarthritis and oligoarthritis (*p* > 0.05) (Table 2).

**Table 2:** Concentration of nitrite/nitrate (NO_2_^−^/NO_3_^−^), thiobarbituric acid-reactive substance (TBARs), and the ferric reducing ability of plasma (FRAP) in sera of children with JIA and the control group

	Children with JIA (*n* = 34)	Children from control group (*n* = 20)	*p* value
NO_2_^−^/NO_3_^−^ (μmol/L)	9.59 ± 4.14	5.59 ± 2.25	0.00069
TBARs (μmol/L)	0.59 ± 0.2	0.61 ± 0.18	0.836
FRAP (mmol/L)	0.75 ± 0.1	0.89 ± 0.17	0.048

A positive correlation was observed between NO_2_^−^/NO_3_^−^ concentrations and JADAS-27 and ESR (*R* = 0.542, *p* = 0.039; *R* = 0.621, *p* = 0.009).

### Determination of TBARs

There were no statistically significant differences in TBAR levels between children with JIA and the control group (0.59 ± 0.2 and 0.61 ± 0.18 μmol/L, respectively) (Table 2). No statistically significant difference was observed in serum TBAR levels between children with polyarthritis and oligoarthritis (*p* > 0.05).

TBARs did not correlate with either clinical or laboratory variables of disease activity.

### FRAP assay

**Fig. 2:**
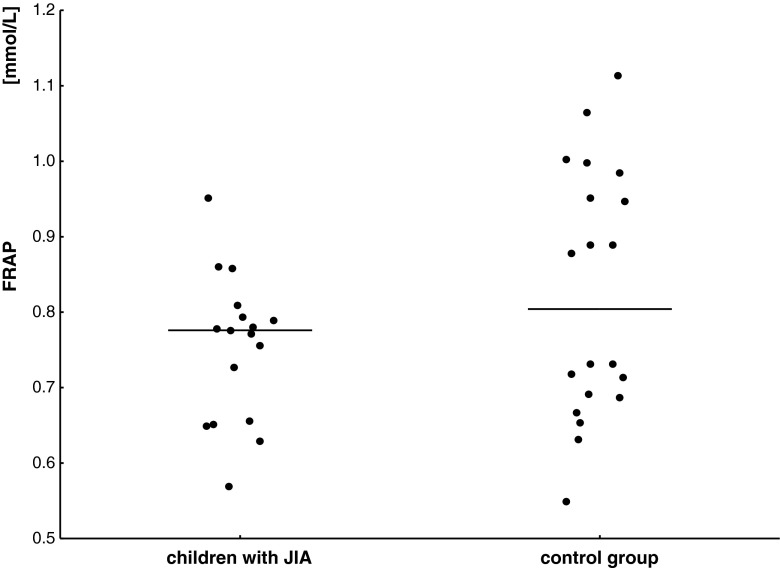
FRAP serum concentration and mean value (*horizontal line*) in children with JIA and in the control group

In the group of children with JIA, FRAP was lower (0.75 ± 0.1 mmol/L) than in healthy subjects (0.89 ± 0.17 mmol/L, *p* = 0.048) (Fig. 2). However, no statistically significant difference was observed in serum FRAP levels between children with polyarthritis and oligoarthritis (*p* > 0.05).

Furthermore, statistically significant negative correlations between WBC vs. FRAP levels (*R* = −0.583, *p* = 0.013) as well as CRP vs. FRAP levels (*R* = −0.633, *p* = 0.0064) in the group of children with JIA were detected.

No statistically significant correlations were detected between distribution of NO end-products, TBARs, and FRAP according to sex, age, and treatment.

## Discussion

Proinflammatory factors such as cytokines and prostaglandins are released at inflammation sites together with ROS [15]. Several studies in patients with RA have documented evidence for increased endogenous NO synthesis, suggesting that overproduction of NO in an inflamed joint may be important for the pathogenesis of RA, but in JIA its role is still unclear [5–9, 16]. To the best of our knowledge, this is the first study assessing the NO end-products nitrite/nitrate, the index of lipid peroxidation (TBARs), and the total antioxidant plasma capacity (FRAP) in children with JIA.

The currently reported data with adult RA patients and confirmed in animal models indicated that NO may function as a disease marker and as a proinflammatory mediator in arthritis [6–9]. Regarding the pathogenesis of RA, it seems that excessive oxidative stress in the inflamed joints is reflected as an increased concentration of NO in peripheral blood [16]. Our study demonstrated a statistically significant increase in serum concentration of NO_2_^−^/NO_3_^−^ in children with JIA compared to healthy subjects, which is in concordance with results obtained by Lotito et al. and Beri et al. [17, 18].

Similarly to other studies, there was no significant difference in NO levels among two JIA subtypes (poly- and oligoarthritis) in our study group [18–20].

Similarly to Bica et al. and Lotito et al., we showed that the concentration of NO end-products in sera of children with JIA and active disease was higher than that in children with inactive process, however without a statistically significant difference [3, 17]. Our study indicated a significant positive correlation between JADAS-27 and ESR and NO end-products’ levels, which is in concordance with previous authors’ observations in adult patients with RA; however, in JIA such correlations were not found [3, 9, 18]. On the other hand, it was observed by Bica et al. that patients with active JIA and erosive disease had significantly higher NO levels, whereas children with an inactive rheumatoid process had similar NO levels regardless of whether erosions were present or not [3].

There is no compliance among authors about the serum concentration of lipid peroxidation products measured as TBARs in children with JIA. However, it is well established that overproduction of ROS in pathological processes, like chronic inflammation, could result in oxygenation of cell membrane compounds (protein structure damage, lipid oxidation), thus inducing loss of cell integrity and functional alteration of cell receptors and enzymes [15]. In our group of children with JIA, no statistically significant difference was detected in the concentration of TBARs either between the two JIA subtypes and healthy subjects, which is in line with previous observations of other authors [20]. In contrary, in another study, increased TBARs were found in sera of children with JIA [21]. Ramos et al. also observed such correlations; however, the authors determined different products of lipid peroxidation than we did [19]. Guney et al. who determined malonodialdehyde (MDA) by thiobarbituric acid test indicated higher MDA levels in patients with JIA, with the highest concentration in systemic JIA [15]. The discrepancies between studies may be also explained by the fact that TBARs are not a very specific marker, and perhaps it would be better to determine specific substances like MDA and lipoperoxide (LPO). Furthermore, patients with rheumatoid process have defective defense mechanisms against ROS [6–9, 22, 23]. It was also proved that inflammatory immune response is upon aging [4, 24]. Perhaps, it could be speculated that similar levels of TBARs in healthy children and those with JIA could be explained by high efficiency of the antioxidant system in children, as well as higher cell membrane stability in young subjects and less susceptibility of the membrane’s components to damaging factors [15].

It is worth noticing that this is the first study on FRAP in children with JIA. Among various methods, assessing the antioxidant capacity of plasma FRAP, which estimates the total antioxidant plasma capacity, seems to be the best, as it gathers whole scavenging abilities of plasma. It is still undecided if a lower level of FRAP is the cause or effect of rheumatoid process. Sarban et al. have described decreased levels of the antioxidant plasma capacity assayed in terms of FRAP in RA patients and postulated that decreased FRAP could contribute to the pathogenesis of rheumatoid process [23]. Although there was no statistically significant difference in FRAP levels, comparison of our study and control groups indicated that FRAP was lower in sera of children with JIA. Perhaps, a small difference between FRAP levels in the study and control groups was due to the efficient defense against ROS in young patients with JIA or an effective anti-inflammatory therapy of JIA that results in adaptive increase of FRAP [25]. Although there was no significant correlation of FRAP with disease activity, it should be underlined that FRAP negatively correlates with CRP and WBC in children with JIA.

## Conclusions

The results provide some evidence for a potential role of the increased NO and decreased antioxidant plasma capacity in the pathogenetic mechanism of JIA. Measurement of NO and FRAP may provide a useful tool to aid in the assessment of the patient’s oxidative stress status and in the determination of an appropriate treatment management plan. Further studies are needed to determine the exact role of ROS and particularly NO in the pathogenesis of JIA and to show its usefulness as a marker of disease activity.
